# Prognostic factors associated with quality of life in heart failure patients considering the use of the generic EQ-5D-5L™ in primary care: new follow-up results of the observational RECODE-HF study

**DOI:** 10.1186/s12875-021-01554-1

**Published:** 2021-10-13

**Authors:** Sigrid Boczor, Marion Eisele, Anja Rakebrandt, Agata Menzel, Eva Blozik, Jens-Martin Träder, Stefan Störk, Christoph Herrmann-Lingen, Martin Scherer, for the RECODE-HF study group, Winfried Adam, Cassandra Behrens, Eva Blozik, Sigrid Boczor, Marion Eisele, Malte Harder, Christoph Herrmann-Lingen, Agata Menzel, Dagmar Lühmann, Anja Rakebrandt, Koosje Roeper, Martin Scherer, Stefan Störk, Jens-Martin Träder

**Affiliations:** 1 Department of General Practice and Primary Care, University Medical Center Hamburg-Eppendorf, Martinistraße 52, 20246 Hamburg, Germany; 2 Department of Primary Medical Care, University of Luebeck, Luebeck, Germany; 3 Comprehensive Heart Failure Center Würzburg, University and University Hospital Würzburg, Würzburg, Germany; 4 University of Göttingen Medical Center and German Center for Cardiovascular Research (DZHK), partner site Göttingen, Göttingen, Germany

**Keywords:** Primary care, Heart failure, Quality of life, EQ-5D-5L

## Abstract

**Background:**

The implementation of care concepts fitting the needs of patients with chronic heart failure (HF) remains challenging. In this context, psycho-emotional well-being is not routinely assessed, and under-researched despite indications that it is of great relevance for, e.g., acceptance, adherence, and prognosis. The aim of this study was to observe clinical characteristics for their prognostic utility in HF patients, and to compare the patients’ health-related quality of life (QoL) with German population norm values.

**Methods:**

The current post-hoc analysis was performed on data collected amongst participants of the RECODE-HF study who had fully answered the EQ-5D-5L™ items at both baseline and 12 months (*n* = 2354). The status in the patients’ self-assessment items, EQ-5D visual analog scale (VAS) and EQ-5D index was categorized into worse/unchanged/improved. General linear mixed models (GLMM) with logit link were applied. Subgroups included 630 patients (26.8%) screened positive and 1724 patients (73.2%) screened negative for psychosocial distress (PSD).

**Results:**

The 12-months change in EQ-5D index, generally resulting from change in individual EQ-5D items, additionally associated not only with high NYHA class but sociodemographics (employment/living alone/GP practice years) (96.2% correctly classified in GLMM). The 12- months change in individual QoL aspects showed associations with age*NYHA, gender, body-mass index, and comorbidities dyslipidemia, myocardial infarction, asthma/chronic pulmonary disease. Important social roles were reflected in particular when HF patients lived alone or the doctor mentioned to the patient that the patient had HF. Patients with/without PSD differed in some sociodemographic and clinical parameters. However, no influence of PSD could be demonstrated in the 12-month follow-up of the EQ-5D-5L™. Nonetheless, comparison of the 12-months QoL with general German population norm values by age groups < 75 years and 75+ showed markedly health restrictions in HF patients in all EQ-5D-5L™ aspects.

**Conclusion:**

Our analysis revealed different prognostic factors primarily associated with change of burden in different QoL aspects in HF patients. In GP practice it is important to consider in addition to the overall day-related VAS all the individual health-related QoL aspects to take a holistic view of the patient, as well as to pay particular attention to the interrelation of individual characteristics.

**Supplementary Information:**

The online version contains supplementary material available at 10.1186/s12875-021-01554-1.

## Background

Chronic heart failure (HF) is a syndrome affecting an estimated 64.3 million people worldwide [1], and predominantly individuals at advanced age [2], but trends also show an increasing number of cases in the relatively young [1]. HF has become a major health problem due to its rising prevalence and associated health care costs [2]. The prevalence of known HF of the general adult population is estimated at 1 to 2% in developed countries and even at 4.2% by a recent meta-analysis on echocardiographic screening studies including beforehand unrecognized cases [1]. Despite improved treatment options, HF still has a very high mortality and re-hospitalization risk, and is characterized by substantial symptom burden and compromised health-related quality of life (QoL) [2]. The EQ-5D in its new 5-level version (EQ-5D-5L™ [3];) has been shown suitable for routinely assessing health-related QoL in patients with HF [4]: Patients with a higher degree of HF severity scored lower in the EQ-5D-5L™ items and the EQ visual analog scale (VAS) [4]. Recently, we reported that the presence of psychosocial distress (PSD) in patients with HF is frequent, affects their QoL as assessed by the EQ VAS, and influences their general practitioners’ (GP) awareness regarding treatment, as well as their medication adherence [5, 6]. As such, psychosocial distress in patients with HF is a problem GPs should regularly pay attention to [2, 7, 8]. Socioeconomic and sociodemographic parameters have also come more into focus with regard to the health-related status in HF patients and require further research in this context [9].

The aim of the analyses presented here is to investigate the prognostic utility of clinical characteristics including psychosocial distress as well as sociodemographic parameters amongst patients studied in the context of RECODE-HF, a prospective cohort study reporting the profile of patients with HF treated at their GPs. We investigated whether prognostic factors for a change in the subjective course of HF could be identified regarding QoL as it is reported by the EQ-5D-5L™, a self-reported measure of patient health-related QoL, which we consider to be an implication for the care of HF patients. For this purpose, we compared different measures of QoL, namely the EQ VAS, the EQ-5D index and the various dimensions included in the EQ-5D-5L™. Since the importance of classifying activities of daily living, mobility and self-care in HF patients has been emphasized in recent research [4], we differentiated the course of HF in individual calculation models for the different aspects of quality of life. Furthermore, we compared EQ-5D-5L™ ratings after 12-month in RECODE-HF patients to the German population norm.

## Methods

HF patients had been recruited to the RECODE-HF study via primary care practices in Germany between 2012 and 2014, with 293 out of 4420 invited GPs participating. Two local ethics committees granted ethical clearance. Patient inclusion criteria had been: written informed consent; age ≥ 18 years; documented HF diagnosis within the last 5 years; leastways 1 GP consultation within last 6 months. Exclusion criteria were: dementia; HF patients not being regular patients at participating GP practice. Of 13,830 patients invited to participate, 5385 consented, and 4909 answered the baseline questionnaire of the RECODE-HF study. The patient’s GP was interviewed by phone, especially assessing comorbidities, and 3387 patients resulted eligible for the RECODE-HF study [5, 10, 11].

For the current post-hoc analysis, we included thereof the 2354 patients who completed the fife EQ-5D-5L™ items, at both the baseline and the twelve-month follow-up questionnaire. The New York Heart Association (NYHA [2];) functional class as rated by the patients’ GP was included as physician-based assessment of baseline disease status. For further comparison the NYHA class was categorized into lower (class I/II) and higher symptom burden (class III/IV). Comorbid conditions were identified, and classified by Charlson’s Comorbidity Index [12]. The Lubben Social Network Scale was used to assess familiar relation and extended social network [13], and the General Self-efficacy Scale according to Hinz et al. for measuring the patients’ self-efficacy [14], both indicating a worse condition by lower scoring. Level of education was described using the Comparative Analysis of Social Mobility in Industrial Nations Project (CASMIN) criteria [15]. Migration background was included in the analyses corresponding to Schenk et al. [16].

The psychosocial distress subgroup [PSD(+)] was defined as patients with present symptoms of depression/adjustment disorder and/or anxiety, compared to patients without psychosocial distress [PSD(−)]. The hierarchical screening algorithm for PSD [11, 17] is founded on the Patient Health Questionnaire Depression Scale (PHQ-9, [18]), the subscales anxiety and depression of the Hospital Anxiety and Depression Scale (HADS-A and HADS-D [19, 20];) and selected items of the PROMIS Anxiety Scale (PROMIS Anxiety [21, 22]). Depressed mood was defined by symptoms of depression/adjustment disorder (PHQ-9 score > 8 and HADS-D score > 8) and anxiety by a PROMIS Anxiety score > 18 [11]. Variables on psychiatric history or treatment were not included in the current analyses since an earlier analysis of RECODE-HF patients with a psychiatric history, psychological or psychiatric treatment showed that ““neither the general practitioner’s awareness of depressive symptoms nor the treatment of depression was significantly associated with the patients’ HRQOL”” [5].

The key elements of the EQ-5D-5L™ are five dimensions (mobility; self-care; usual activities; pain/discomfort; anxiety/depression) related to the patient’s well-being by a descriptive system. The patients answer on a 5-point scale (no/slight/moderate/severe/extreme problems), resulting in a 5-digit number describing the patient’s state of health. The second EQ-5D-5L™ measure is the EQ visual analogue scale, whereby the patient is rating his/her actual well-being with a number ranging from 0 to 100, with lower values indicating a worse quality of life. The difference of the 12-month minus the baseline EQ VAS value was calculated. Thus positive values indicate an improved actual feeling of well-being while negative values indicate a worsened feeling at the 12-month follow-up in comparison to the baseline status. The EQ-5D descriptive system and the EQ VAS are measurements of different conceptual ideas not depicting exactly the same [23–25]. This means an improvement (or deterioration) in one of the items of the EQ-5D-5L™ does not necessarily show up as an improvement (or deterioration) in the EQ VAS.

EQ-5D index calculation was based on the EuroQoL Groups’ crosswalk calculation [26]. This is despite the fact that the German value set on the EQ-5D-5L™ was recently published [27], to make results comparable to recent EQ-5D-5L™ validation analyses using the crosswalk index. No missing data imputation was performed, rather only questionnaires with complete EQ-5D-5L™ items for baseline and follow-up were included in the analyses.

Statistical analyses were performed with IBM SPSS for windows version 25.

### Statistical analysis

Patient baseline characteristics and comorbid conditions are presented as mean and standard deviation (SD) for continuous data, and absolute and relative frequencies for categorical data, in total, and stratified by PSD. Baseline and follow-up QoL parameters are presented as mean and SD for the EQ VAS and EQ-5D index, and absolute and relative frequency for each EQ-5D-5L™ item, respectively, stratified by PSD and NYHA classification. The twelve-month change in the QoL parameters is shown by mean difference and 95% confidence interval (CI) for the EQ VAS and EQ-5D index, and the odds ratio for having vs. not having any health-related QoL problem after 12 months of follow and 95% CI for the EQ-5D-5L™ items. Additionally, for each EQ-5D-5L™ item the percent of patients reporting health-related QoL problems at 12-month visit, but without respective problem at baseline is given, based on the number of patients without health-related QoL problems at baseline.

Baseline outcomes between PSD(+) and PSD(−) patients were compared using the Student t-test and chi-squared test, as appropriate. For comparison of the change in EQ VAS and EQ index between baseline and follow-up, respectively, paired t-tests were calculated, and the McNemar test was used for the ordinal EQ-5D-5L™ items summarized into any vs. no problem. Comparisons were done in strata for lower vs. higher NYHA functional class and PSD group, respectively.

The individual change per patient in health-related QoL baseline to 12-month follow-up was recoded into worse/unchanged/improved condition. To analyze the relationship of change with possible influencing factors, generalized linear mixed models (GLMM) were used with the change as dependent variable for each EQ-5D-5L™ item, the EQ VAS and EQ German index, respectively. The multinomial distribution with logit type link for ordinal dependent variables (cumulative) was used. The goal of the GLMM was to determine the effect of independent variables on the probability of obtaining a change in one of the health-related QoL aspect models at 12-month follow-up, which could be an increasing or decreasing condition. To compare the fit between the absence or presence of a particular variable, the change in likelihood was used [28]. Fixed effects included NYHA functional class and PSD group in all models. Other factors considered that suggested a difference in outcome measures were a) general descriptors as gender, age, body mass index, type of health insurance, household net income, employment status, living alone, the CASMIN education classification, and migration background; b) factors characterizing the GP, i.e. years of GP practice, additional psychotherapeutic training of GP; c) comorbidities with prevalence greater than 20% in the total patient sample, i.e. cardiac decompensation, arterial hypertension, chronic ischemic heart disease, dyslipidemia, myocardial infarction, chronic pulmonary disease; d) further items characterizing QoL and the patient’s self-perception including the question ‘Has a doctor ever told you that you have HF?‘, EQ VAS at baseline, the score in the Lubben Social Network Scale, the General Self-efficacy Scale, EQ-5D-5L™ baseline assessments, and change at follow-up in the other EQ-5D-5L™ parameters.

We analysed whether patients screened positively for PSD at baseline would show more problems regarding mobility, self-care, usual activities, pain/discomfort, and anxiety/depression as indicated by the five EQ-5D-5L™ items compared to negatively screened patients, at baseline as well as at 12-month follow-up.

We further analysed whether presence of psychosocial distress or other clinical criteria could add prognostic value to the course of HF as indicated by a change in health-related QoL aspects assessed by the EQ-5D-5L™, i.e. the individual items, EQ VAS, and EQ-5D index.

For comparison with general population norm values [29] dichotomized EQ-5D-5L™ frequencies were used (any/no problems), as well as the EQ VAS and EQ index. Calculations were performed by the one-sample chi-squared test in case of frequencies, and the one-sample t-test in case of continuous parameters, respectively, using the population norm value as test value.

## Results

**Fig. 1:**
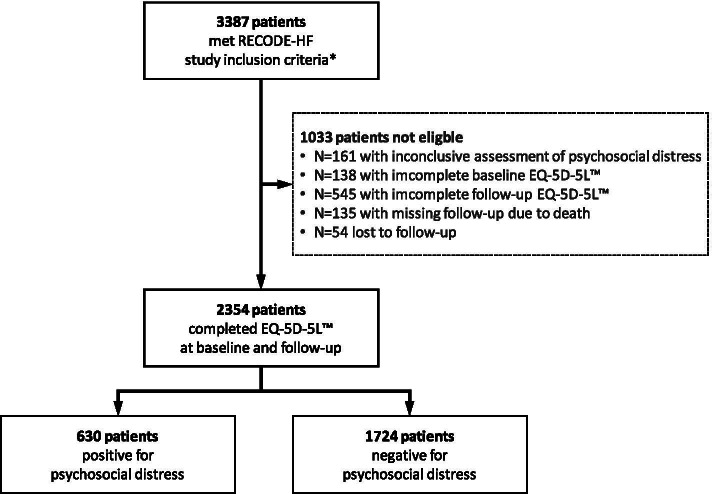
Sampling frame. *A detailed description of the RECODE-HF study criteria can be found here [3]

A flow-chart showing the patient sample for this study is presented in Fig. 1.

Applying the above mentioned definitions, we found that out of 2354 patients with complete EQ-5D-5L™ *n* = 630 (26.8%) suffered from psychosocial distress, PSD(+). This included *n* = 356 patients with depressed mood and *n* = 274 with anxiety. Accordingly, *n* = 1724 (73.2%) were considered PSD(−).

Patient baseline characteristics are shown in Table 1 for the total sample and by psychosocial distress, missing rates were similar.

**Table 1:** Patient baseline characteristics of the total sample and in subgroups of presence of psychosocial distress

	All patients ***N*** = 2354	PSD(+) N = 630	PSD(−) N = 1724	***P*** value
Gender				< 0.001
Female	1035 (44.0%)	334 (53.8%)	701 (41.2%)	
Male	1288 (54.7%)	287 (46.2%)	1001 (58.8%)	
Missing	31 (1.3%)	9 (1.4%)	22 (1.3%)	
Age in years, mean ± SD	73.2 ± 10.0	71.9 ± 10.9	73.7 ± 9.6	< 0.001
Missing	117 (5.0%)	36 (5.7%)	81 (4.7%)	
> 75 years of age	1125 (47.8%)	277 (44.0%)	848 (49.2%)	0.070
NYHA functional class^a^				0.001
I	589 (25.0%)	135 (21.4%)	454 (26.3%)	
II	1190 (50.6%)	304 (48.3%)	886 (51.4%)	
III	498 (21.2%)	164 (26.0%)	334 (19.4%)	
IV	43 (1.8%)	18 (2.9%)	25 (1.5%)	
Missing	34 (1.5%)	9 (1.5%)	25 (1.5%)	
Body mass index, mean ± SD	29.2 ± 6.0	29.9 ± 6.4	29.0 ± 5.8	0.002
Missing (no calculation possible)	223 (9.5%)	70 (11.1%)	153 (8.9%)	
Currently employed				0.710
Yes	193 (8.2%)	48 (7.6%)	145 (8.4%)	
No	2126 (90.3%)	574 (91.1%)	1552 (90.0%)	
Missing	35 (1.5%)	8 (1.3%)	27 (1.6%)	
Living alone				0.385
Yes	675 (28.7%)	194 (30.8%)	481 (27.9%)	
No	1642 (69.8%)	426 (67.6%)	1216 (70.5%)	
Missing	37 (1.6%)	10 (1.6%)	27 (1.6%)	
Lubben Social Network Scale^b^, mean ± SD	15.3 ± 5.8	13.6 ± 5.9	15.9 ± 5.7	< 0.001
Missing	78 (3.3%)	27 (4.3%)	51 (3.0%)	
General Self-efficacy Scale^c^, mean ± SD	31 ± 6	27 ± 7	33 ± 6	< 0.001
Missing	26 (1.1%)	12 (1.9%)	14 (0.8%)	
Health insurance				0.035
Statutory	2092 (88.9%)	577 (91.6%)	1515 (87.9%)	
Private	190 (8.1%)	35 (5.6%)	155 (9.0%)	
Social welfare agency	14 (0.6%)	8 (0.8%)	9 (0.5%)	
Missing	58 (2.5%)	13 (2.1%)	45 (2.6%)	
Household net income per capita, mean (SD) in Euro	1167 ± 562	1070 ± 543	1202 ± 565	< 0.001
Missing	474 (20.1%)	130 (20.6%)	344 (20.0%)	
Education level (CASMIN)				< 0.001
Primary	1473 (62.6%)	439 (69.7%)	1034 (60.0%)	
Secondary	636 (27.0%)	145 (23.0%)	491 (28.5%)	
Tertiary	39 (6.2%)	39 (6.2%)	169 (9.8%)	
Missing	7 (1.1%)	7 (1.1%)	30 (1.7%)	
Migration background^d^				0.001
Yes	131 (5.6%)	53 (8.4%)	78 (4.5%)	
No	2082 (88.4%)	536 (85.1%)	1546 (89.7%)	
Missing	141 (6.0%)	41 (6.5%)	100 (5.8%)	

Data are mean ± standard deviation (SD) or n (%), as indicated

*PSD* Psychosocial distress (classification according to hierarchical algorithm; Eisele et al. 2017), *NYHA* New York Heart Association, *CASMIN* Comparative Analysis of Social Mobility in Industrial Nations Project (CASMIN criteria)

^a^Assessed by general practitioner. ^b^According to Lubben et al. 2006. ^c^According to Hinz et al. 2006. ^d^According to Schenk et al. 2006

PSD(+) patients were more often women, 2 years younger on average, and had a higher symptom burden. PSD(+) patients with lower vs. higher NYHA class were more often younger than 75 years, while this difference was not apparent for PSD(−) patients. PSD(+) patients had a higher body mass index, and scored lower at the Lubben Social Network Scale and the self-efficacy score. Educational and income level in PSD(+) patients was lower, and they had more often a migration background. The respective differences regarding comorbidities are presented in Table 2.

**Table 2:** Charlson comorbidity index^a^ and patient comorbidities^b^ in total and by presence of psychosocial distress

	All patients N = 2354	PSD(+) N = 630	PSD(−) N = 1724	***P*** value
Charlson comorbidity index				0.600
0	118 (5.0%)	25 (4.0%)	93 (5.4%)	
1–2	1012 (43.0%)	270 (42.9%)	742 (43.0%)	
3–4	585 (24.9%)	156 (24.8%)	429 (24.9%)	
≥ 5	217 (9.2%)	64 (10.2%)	153 (8.9%)	
No calculation possible	422 (17.9%)	115 (18.3%)	307 (17.8%)	
Cardiac decompensation or congestive heart failure with dyspnea improving during therapy	1737 (73.8%)	458 (72.7%)	1279 (74.2%)	0.702
Arterial hypertension	1175 (49.9%)	309 (49.0%)	866 (50.2%)	0.611
Chronic ischemic heart disease (also after myocardial infarction, ischemic cardiomyopathy, angina pectoris)	849 (36.1%)	226 (35.9%)	23 (36.1%)	0.906
Dyslipidemia	565 (24.0%)	154 (24.4%)	411 (23.8%)	0.761
Myocardial infarction	523 (22.2%)	135 (21.4%)	388 (22.5%)	0.483
Asthma/chronic pulmonary disease with pulmonary dyspnea	497 (21.1%)	164 (26.0%)	333 (19.3%)	0.002
Has a doctor ever told you that you have heart failure?				0.040
Yes	1725 (73.3%)	485 (77.0%)	1240 (71.9%)	
No	443 (18.8%)	9 (15.4%)	346 (20.1%)	
I do not know anymore	157 (6.7%)	43 (6.8%)	114 (6.6%)	
Missing	29 (1.2%)	5 (0.8%)	24 (1.4%)	

^a^According to Charlson et al. 1987. ^b^All comorbidities occurring at prevalence > 20% were listed

*PSD* Psychosocial distress classified according to hierarchical algorithm; Eisele et al. 2017

PSD(+) patients presented more often with asthma (*p* = 0.002), and had more often been told by a doctor that they suffered from HF.

**Fig. 2:**
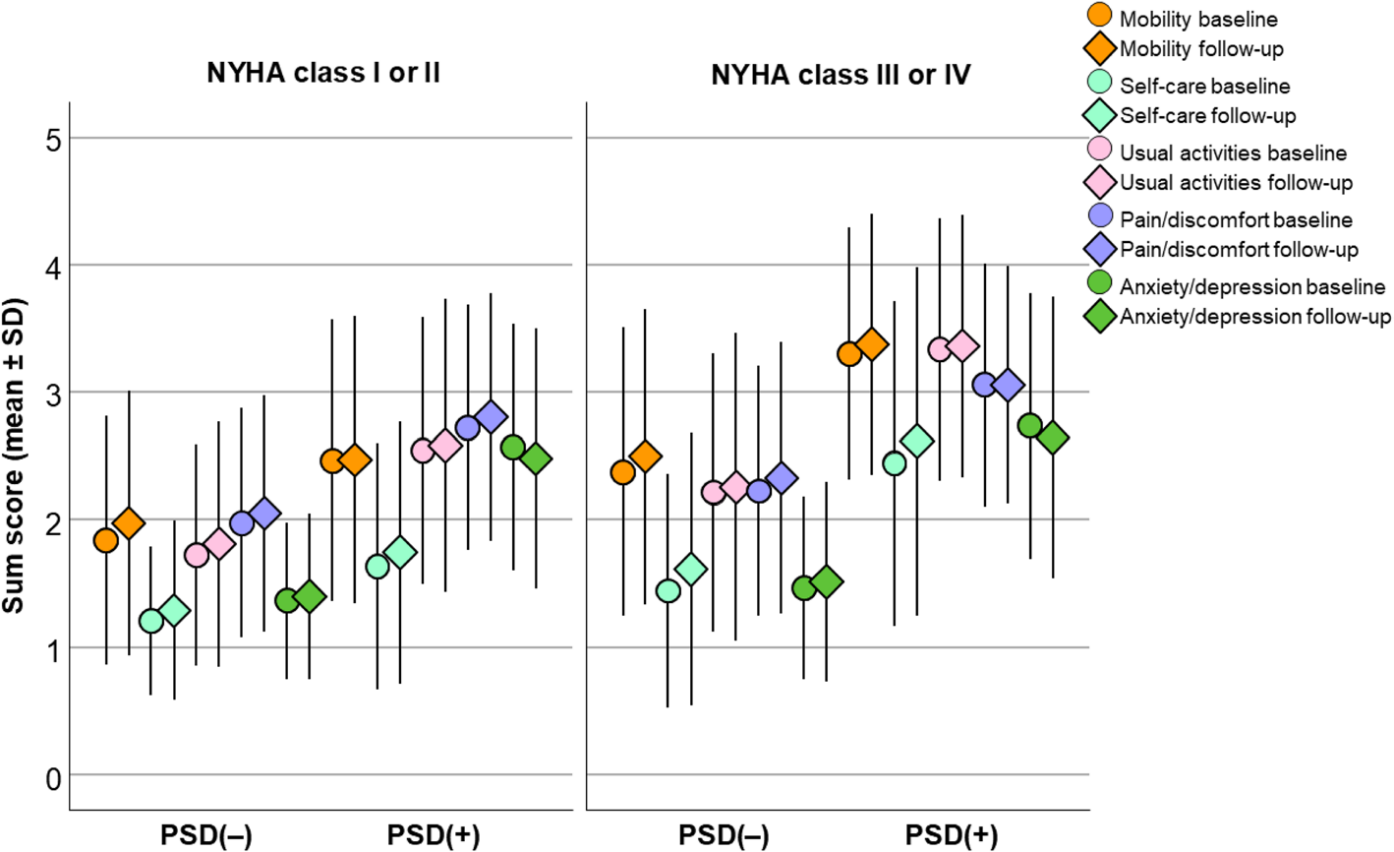
Patient impairment represented by EQ-5D-5L™ items at baseline and follow-up by HF severity* and PSD**. The patient performance as expressed by EQ-5D-5L™ measured health-related quality of life at baseline and 12-month follow-up, by severity of heart failure and presence of psychosocial distress (PSD). Score values on the y-axis correspond to categories derived from EQ-5D-5L™ items: 1 = no problem, 2 = slight, 3 = moderate, 4 = severe, 5 = extreme problems. PSD(−) = Psychosocial distress absent; PSD(+) = Psychosocial distress present; PSD classification according to hierarchical algorithm (for details see Methods). NYHA = New York Heart Association functional class as assessed by the patients’ general practitioner at baseline; SD = standard deviation; N total = 2320

Figure 2 shows using mean sum scores of EQ-5D-5L™ items the patients’ impairment represented in the PSD(+) vs. PSD(−) group and by NYHA category at baseline and 12-month follow-up.

It is apparent that the higher score levels in NYHA functional class III/IV vs. class I/II patients were particularly pronounced by additional presence of psychosocial distress. All health-related QoL measures differed markedly by NYHA classification (low vs. high) and presence of psychosocial distress both at baseline and after 12 months (all *p* <  0.001; Table 3).

Comparing the odds ratios of any health-related QoL problem vs. no QoL problem at 12-month follow-up to baseline status, similarly in higher and lower NYHA class mobility and pain/discomfort (both EQ-5D dimensions only in low NYHA), and self-care differed markedly in PSD(−) but not in PSD(+) patients (Table 4).

No marked differences were present in usual activities, while anxiety/depression presented a marked difference in PSD(+) patients with low NYHA. Mean differences in the EQ VAS and German index were markedly higher in PSD(−) but not in PSD(+) patients.

The best GLMM (96.2% correct classified in total) was the EQ index change model associating with high NYHA class, employment, living alone, GP practice years, a change in EQ-5D-5L™ items mobility, self-care, usual activities, pain/discomfort, and anxiety/depression, as well as baseline anxiety/depression and pain/discomfort. Age, body-mass index, a change in usual activities and anxiety/depression, and baseline mobility, usual activities, and pain/discomfort revealed as associated factors for change in mobility; age*NYHA, dyslipidemia, baseline status of all EQ-5D-5L™ items except pain/discomfort, if a doctor had ever told the patient having HF, and a change in usual activities, pain/discomfort, anxiety/depression for change in self-care; myocardial infarction, baseline VAS, mobility, usual activities, pain/discomfort, and a change in mobility, self-care, and pain/discomfort for change in usual activities; chronic pulmonary disease, living alone, baseline mobility, pain/discomfort, anxiety/depression, and a change in usual activities for change in pain/discomfort; gender, baseline usual activities and anxiety/depression, a change in mobility, usual activities, and pain/discomfort for change in anxiety/depression.

Compared to related age groups of the German general population norm, frequencies of any problem were markedly higher with regard to all EQ-5D-5L™ items in our HF patients, correspondingly the mean German index and the EQ VAS were lower (Table 5).

Self-care at an age up to 74 years showed with − 12% the lowest decrease compared to the norm value. Moreover it was the only parameter in which difference to the norm almost doubled in the elder patient group 75+ while the other items showed similar deviations regarding age groups. In both age groups the highest deviation from the norm was present in usual activities (− 35%; − 36%) and anxiety/depression (− 36%; − 40%). The younger HF patients without PSD differed markedly from the population norm in the EQ index (*p* <  0.001) as well as the younger HF patients with PSD who also differ in the EQ VAS (both *p* < 0.001). Also the 75+ HF patients with PSD differed from the population regarding EQ VAS and index (both *p* < 0.001). Differentiated by PSD only younger patients ≤74 yrs. without PSD showed EQ VAS values similar to the population norm (*p* = 0.084; *n* = 768). Elderly patients of 75+ without PSD had a lower mean EQ VAS (*p* = 0.036) than the norm but did not differ by the EQ German index (*p* = 0.787; *n* = 848).

## Discussion

Our analyses revealed that a change of burden in health-related quality of life aspects in HF patients was not primarily associated with psychosocial distress but different clinical characteristics. Nevertheless, our analyses confirmed that HF patients with PSD showed higher rates in problems with mobility, self-care, usual activities, pain/discomfort and anxiety/depression as self-reported by the EQ-5D-5L™ at baseline and 12 months of follow-up, respectively, than HF patients without PSD. In our RECODE-HF patient sample as a whole, health-related QoL problems were assessed as markedly higher than in the German population norm among all EQ-5D-5L™ dimensions as well as on the EQ VAS.

### The complexity of psychosocial distress, comorbidities, sociodemographics, and quality of life

In contrast to other studies [30, 31] we did not find psychosocial distress as a prognostic factor for the deterioration of health in HF patients, which might be due to differing definitions of a depressive symptomatology. Byrne et al. 2018 observed depression in order to predict all-cause mortality in HF patients [30]. Unlike Byrne et al. we did not observe mortality but the change in health-related QoL over time course as proxy for a deterioration of health. While depression severity in the study of Byrne et al. did not predict all-cause mortality, depression symptoms, hopelessness and cognitive impairment did predict it [30]. In our RECODE-HF study cognitive impaired patients were excluded. Hare et al. 2013 concluded from their HF patients’ study that depression seems to predict more strongly health-related QoL than sociodemographic or lifestyle issues, NYHA class or co-morbidities; conversely, social factors and health status of HF patients with poor health-related QoL on the Kansas City Cardiomyopathy Questionnaire could predict the development of depression [32]. Recently we identified social factors as well as somatic comorbidities associated with a loss of medication and lifestyle adherence in HF patients [6, 33]. Kashem et al. 2019 analysed data from a population-based Canadian survey and found deprivation associated with worse health-related QoL as assessed by the EQ-5D-5L™, and recommended poverty reduction strategies [34]. This point can be emphasized by our analyses which showed that HF patients with psychosocial distress reported lower income thus suggesting they had to cope with an additional quality of life burden. Our PSD(+) patients showed less a private insurance and correspondingly lower income, lower income in turn increases existing stress that is already due to HF burden. Social factors and health status had previously been found predictive of an onset of depressive symptoms in HF patients [31]. We agree with others who recommend a specialised risk assessment and management strategies for depressive symptomatology in HF patients [30–32, 35]. Our PSD(+) patients differed markedly from the PSD(−) group, as reported earlier [5, 36]. Our current post-hoc analyses on 2354 RECODE-HF study patients identified among PSD(+) and PSD(−) patients clinical and sociodemographic factors associated with a change in varying health-related QoL parameters after twelve months. Our findings fit Verma et al. 2017, who reported a higher socioeconomic status as well as having a partner associating with a higher quality of life at baseline and with a higher functional capacity in a post-hoc analysis of the HF-ACTION study [9]. However, Verma et al. 2017 could not affirm them as independent predictors of long-term clinical outcomes over a median follow-up of 2.5 years, and recommended further research in these results [9]. We were able to show living alone as important sociodemographic factor in our current post-hoc RECODE-HF analyses, as well as the social role in the doctor’s mentioning to the patient that the patient had HF.

### Patient functional capacities and quality of life

Boczor et al. 2019 stressed the importance of risk factors for quality of life in HF patients, especially the differentiation of health-related QoL by the severity of HF [4]. In this new analysis, especially mobility, self-care and usual activities were found to be prognostic factors associated with a change in EQ-5D-5L™ items at 12-months follow-up. Based on results by Eisele et al. 2017 that the baseline EQ VAS showed to be different in PSD(+) patients with regard to the GP’s awareness of psychosocial distress, we further investigated how patient subgroups with and without PSD differ in HF patients. Here we included patients with anxiety which were excluded by Eisele et al. in their more specified analyses regarding depression treatment. As could be verified by Boczor et al. 2017 [37], the NYHA functional class is predictive for health-related QoL in HF patients, and was included in our present analyses as influence factor. Accordingly as presented in Table 4, the individual 12-month change confirmed a higher increase in any health-related QoL problem, defined as patients who did not report the respective problem at baseline, from 33% up to 72% in PSD(+) patients with higher NYHA burden compared to 17% up to 57% with a lower NYHA burden, respectively. Highest increase rates during our follow-up interval of 1 year were reported due to pain/discomfort, also in PSD(−) patients (ranging from an increase of 9 to 37% in low and 15 to 36% in higher NYHA class).

We found the EQ index change especially associating high NYHA, employment status, and living alone HF patients, also years of GP practice. Besides age, especially a change in usual activities, and anxiety/depression were associated factors for a change in mobility; as were especially dyslipidemia and interestingly if a doctor had ever told the patient having HF for a change in self-care. Myocardial infarction and change in mobility, self-care, and pain/discomfort associated for change in usual activities; chronic pulmonary disease, living alone, and a change in usual activities for the change of pain/discomfort; gender and a change in mobility, usual activities, and pain/discomfort for a change of anxiety/depression. The influential characteristics should be followed up conjointly in HF patients by the GP in primary care practices.

### Comparison with general population

In our HF patients health-related QoL problems were overall markedly higher than in the German population norm [29], among all EQ-5D-5L™ dimensions. Our data underline the importance of treating health-related QoL problems in HF patients, in those below 75 years of age as well as in the elderly, and with a special focus on psychosocial distress. The complexity of QoL especially in relation to severe disease burden as present in heart failure and PSD needs to be addressed in further research and health care services. The lifetime risk of developing heart failure at age 55 years was recently reported as 33% for men and 28% for women, and as concerning ≥10% among people > 70 years of age [2]. Horackova et al. 2019 presented the prevalence of late-life depression ranging from 17 to 35% across European regions and reported a “gap in mental health service use” in those countries [35]. Llaquet Bayo et al. 2019 analysed in their Spanish follow-up study parameters associated with a worse health-related quality of life assessed by the EQ-5D-5L™ in 200 major trauma patients [38]. They reported an age greater or equal 55, female gender and unskilled employment found to be risk factors for a lower EQ VAS; female gender was most importantly associated with pain and – consistent with our analysis with depression/anxiety. Age greater or equal 55 years, female gender and severe injury were associated with a lower EQ. 5D index value. Llaquet Bayo et al. concluded that despite an improvement of health-related QoL in the first year after major trauma patients did not regain normal reference levels.

We assume that younger HF patients ≤74 yrs. without PSD were similar experiencing their actual daily state of health in general compared to the population norm, while younger PSD(+) patients differ due to their additional PSD burden. Nevertheless, we found younger HF patients without PSD differing in the EQ index, i.e. the health-related QoL aspects of the EQ-5D-5L™ as a whole (*p* < 0.001). This implies these aspects need to be looked at in detail in HF patients even if not showing any PSD. Our analyses confirmed that elderly HF patients (75+) without PSD show a decrease in the current health status in general, as is a known fact influenced by aging. Regarding health-related QoL aspects as a whole as assessed by the EQ-5D-5L™ index, elderly HF patients without PSD show a similar status compared to their age group in normal population [29]. Our analyses showed that also elderly HF patients with PSD need a closer health care as they show a markedly lower VAS and EQ index than the general population norm in their age group 75+ (*p* < 0.001).

### Detecting changes

Peters et al. 2014 conducted a cohort study in 4485 primary care practice patients with different diseases, thereof 520 heart failure patients [39]. They used the EQ-5D 3-level version as well as a disease-specific measure for assessing health-related QoL. The HF patients’ response rate after 1 year was 66.2%. For the EQ-5D 3-level version the only change in their HF patients at follow-up was found in the EQ VAS and in an additional question upon “change in health” in general in the preceding year. In our analysis the rate of completed EQ-5D-5L™ items at baseline and follow-up was 69.5% (*n* = 2354). We agree with Peters et al. that detected changes might have been the result of multiple testing of study data. Peters et al. concluded as well, that patients with a decreasing health status might have been less likely to take part in the follow-up as patients showed with a lower EQ VAS at baseline over all diseases (asthma, chronic obstructive pulmonary disease, diabetes, epilepsy, stroke and heart failure). They also discussed that patient-reported outcome measures showing no change over time might not be sensitive enough to detect a change in a patient condition. Also evidence might differ according to different study and study patients’ conditions including time frame of follow-up and disease status. We analysed a twelve month follow-up time, however the preceding duration of HF had not been assessed at baseline in the RECODE-HF study. So in the comparison with population norm values the patients’ follow-up rates were used. The missing previous duration of HF is a limitation, as because of this, the patients self-assessed baseline values might be influenced due to an underlying different duration of HF. On the other hand the NYHA functional class, which is a confirmed influence factor in health-related QoL burden in HF patients, was only assessed at baseline by the GP. So any worsening in health-related QoL after twelve months could not be analysed regarding a possible change in NYHA class. To our knowledge, the responsiveness of the EQ-5D-5L™ has not yet been confirmed for HF patients. Nevertheless, our results suggested that clinical as well as socioeconomical parameters associated with a change in health-related QoL aspects after twelve months as assessed by the EQ-5D-5L™ could be identified in our HF patients setting.

## Conclusions

The EQ-5D-5L™ index with the best prognostic model for health-related quality of life in HF patients revealed in particular the implication for further research projects that enable calculation of this index rather than in GP practice. Here it is important to consider in addition to the overall image by the VAS all the five health-related QoL aspects to take a holistic view of the patient, as well as to pay particular attention to the interrelation of individual characteristics.

Regarding health-related QoL in HF patients the very short assessment with the EQ-5D-5L™ can be recommended in GP practices (which is not bound to the written form but can also be used as an interview). Primarily with a change of burden associated characteristics in different health-related quality of life aspects should be followed up accordingly, especially to confine a possible deterioration of the overall disease burden of HF patients.

## Supplementary Information

**Additional file 1: Table 3.** Quality of life by the EQ-5D-5L™ at baseline and follow-up by NYHA* and psychosocial distress. Health-related quality of life as assessed by the EQ-5D-5L™ at baseline and follow-up differentiated by low/ high New York Heart Association functional class and presence of psychosocial distress.

**Additional file 2: Table 4.** Twelve-month change in health-related QoL by NYHA functional class and presence of psychosocial distress. Parametrisation of the twelve-month change in EQ-5D-5L elements differentiated by low/ high New York Heart Association functional class and presence of psychosocial distress.

**Additional file 3: Table 5.** EQ-5D-5L™ ratings obtained after 12-month in RECODE-HF patients compared to the German population norm. Comparison of the twelve-month EQ-5D-5L™ ratings to German population norm values as assessed by the EQ-5D-3L (Szende, Janssen and Cabases 2014), age group 65–74 (*n* = 213) and 75+ (*n* = 149) reported, respectively, related to our RECODE-HF patient sample.

## Data Availability

The data from the RECODE-HF study is not available for public use, as the data is owned by the RECODE-HF Study Group and the authors are not allowed to share this information with third parties. The data that support the findings of this research are available from the corresponding author upon reasonable request.

## Abbreviations

CASMIN: The Comparative Analysis of Social Mobility in Industrial Nations Project criteria
CI: Confidence interval
EQ-5D: Standardized instrument developed by the EuroQoL Group as a measure of health-related quality of life
EQ-5D-5L™: 5-level EQ-5D version
GP: General practitioner
GLMM: Generalized linear mixed models
HADS-A: Hospital Anxiety Scale
HADSD: Hospital Depression Scale
HF: Heart failure
NYHA: New York Heart Association
PHQ-9: Patient Health Questionnaire depression subscale
PROMIS®: Patient-Reported Outcomes Measurement Information System
PSD(+): Psychosocial distress present
PSD(−): Psychosocial distress not present
QoL: Quality of life
SD: Standard deviation
VAS: Visual analogue scale
Vs: Versus
Yrs.: Years
