# Supplementary material for: Prognostic factors associated with quality of life in heart failure patients considering the use of the generic EQ-5D-5L™ in primary care: new follow-up results of the observational RECODE-HF study
Source: BMC Fam Pract. 2021 Oct 13;22:202. doi: 10.1186/s12875-021-01554-1 (PMC8515733; doi:10.1186/s12875-021-01554-1)
Supplement: Supplementary file 1 — Additional file 1: Table 3. Quality of life by the EQ-5D-5L™ at baseline and follow-up by NYHA* and psychosocial distress. Health-related quality of life as assessed by the EQ-5D-5L™ at baseline and follow-up differentiated by low/ high New York Heart Association functional class and presence of psychosocial distress. [file 12875_2021_1554_MOESM1_ESM.docx]

**Table 3.** Quality of life by the EQ-5D-5L™ at baseline and follow-up by NYHA* and psychosocial distress

|  | **Quality of life  parameter** | **Subcategory** | **Baseline** | |  | **Follow-up** | |
| --- | --- | --- | --- | --- | --- | --- | --- |
|  |  |  | **PSD(+)** | **PSD(–)** |  | **PSD(+)** | **PSD(–)** |
| **NYHA functional class I/ II, n=1779** |  |  | **N=439** | **N=1340** |  | **N=439** | **N=1340** |
|  | **EQ VAS**, mean ± SD** |  | 50 ± 18 | 67 ± 18 |  | 51 ± 19 | 65 ± 19 |
|  | **EQ German index, mean ± SD** |  | 0.693 ± 0.221 | 0.855 ± 0.145 |  | 0.678 ± 0.230 | 0.834 ± 0.166 |
|  | **Mobility,  n (%)** | No problems  Slight problems  Moderate problems  Severe problems  Extreme problems | 109 (24.8%)  111 (25.3%)  134 (30.5%)  76 (17.3%)  9 (2.1%) | 656 (49.0%)  341 (25.4%)  251 (18.7%)  87 (6.5%)  5 (0.4%) |  | 116 (26.4%)  102 (23.2%)  127 (28.9%)  87 (19.8%)  7 (1.6%) | 594 (44.3%)  326 (24.3%)  294 (21.9%)  116 (8.7%)  10 (0.7%) |
|  | **Self-care,  n (%)** | No problems  Slight problems  Moderate problems  Severe problems  Extreme problems | 270 (61.5%)  95 (21.6%)  47 (10.7%)  19 (4.3%)  8 (1.8%) | 1155 (86.2%)  119 (8.9%)  46 (3.4%)  16 (1.2%)  4 (0.3%) |  | 253 (57.6%)  88 (20.0%)  63 (14.4%)  28 (6.4%)  7 (1.6%) | 1104 (82.4%)  128 (9.6%)  73 (5.4%)  29 (2.2%)  6 (0.4%) |
|  | **Usual activities, n (%)** | No problems  Slight problems  Moderate problems  Severe problems  Extreme problems | 73 (16.6%)  155 (35.3%)  125 (28.5%)  72 (16.4%)  14 (3.2%) | 50.4% (676)  31.3% (420)  14.7% (197)  2.7% (36)  0.8% (11) |  | 89 (20.3%)  129 (29.4%)  120 (27.3%)  78 (17.8%)  23 (5.2%) | 648 (48.4%)  408 (30.4%)  196 (14.6%)  69 (5.1%)  19 (1.4%) |
|  | **Pain/discomfort,  n (%)** | No problems  Slight problems  Moderate problems  Severe problems  Extreme problems | 49 (11.2%)  126 (28.7%)  171 (39.0%)  85 (19.4%)  8 (1.8%) | 474 (35.4%)  513 (38.3%)  273 (20.4%)  76 (5.7%)  4 (0.3%) |  | 42 (9.6%)  116 (26.4%)  182 (41.5%)  83 (18.9%)  16 (3.6%) | 431 (32.2%)  518 (38.7%)  291 (21.7%)  94 (7.0%)  6 (0.4%) |
|  | **Anxiety/depression,  n (%)** | No problems  Slight problems  Moderate problems  Severe problems  Extreme problems | 58 (13.2%)  160 (36.4%)  141 (32.1%)  73 (16.6%)  7 (1.6%) | 944 (70.4%)  315 (23.5%)  72 (5.4%)  9 (0.7%)  0 (0.0%) |  | 77 (17.5%)  158 (36.0%)  132 (30.1%)  60 (13.7%)  12 (2.7%) | 919 (68.6%)  325 (24.3%)  81 (6.0%)  15 (1.1%)  0 (0.0%) |
| **NYHA functional class III/IV, n=541** |  |  | **N=182** | **N=359** |  | **N=182** | **N=359** |
|  | **EQ VAS**, mean ± SD** |  | 40 ± 18 | 60 ± 20 |  | 41 ± 20 | 56 ± 22 |
|  | **EQ German index, mean ± SD** |  | 0.550 ± 0.248 | 0.783 ± 0.196 |  | 0.533 ± 0.271 | 0.751 ± 0.231 |
|  | **Mobility,  n (%)** | No problems  Slight problems  Moderate problems  Severe problems  Extreme problems | 12 (6.6%)  23 (12.6%)  55 (30.2%)  82 (45.1%)  10 (5.5%) | 107 (29.8%)  84 (23.4%)  101 (28.1%)  60 (16.7%)  7 (1.9%) |  | 14 (7.7%)  17 (9.3%)  53 (29.1%)  83 (45.6%)  15 (8.2%) | 95 (26.5%)  84 (23.4%)  94 (26.2%)  79 (22.0%)  7 (1.9%) |
|  | **Self-care,  n (%)** | No problems  Slight problems  Moderate problems  Severe problems  Extreme problems | 59 (32.4%)  35 (19.2%)  51 (28.0%)  23 (12.6%)  14 (7.7%) | 274 (76.3%)  41 (11.4%)  20 (5.6%)  19 (5.3%)  5 (1.4%) |  | 52 (28.6%)  39 (21.4%)  41 (22.5%)  27 (14.8%)  23 (12.6%) | 249 (69.4%)  43 (12.0%)  37 (10.3%)  18 (5.0%)  12 (3.3%) |
|  | **Usual activities,  n (%)** | No problems  Slight problems  Moderate problems  Severe problems  Extreme problems | 9 (4.9%)  31 (17.0%)  50 (27.5%)  74 (40.7%)  18 (9.9%) | 115 (32.0%)  108 (30.1%)  92 (25.6%)  32 (8.9%)  12 (3.3%) |  | 7 (3.8%)  30 (16.5%)  59 (32.4%)  62 (34.1%)  24 (13.2%) | 128 (35.7%)  90 (25.1%)  82 (22.8%)  39 (10.9%)  20 (5.6%) |
|  | **Pain/discomfort, n (%)** | No problems  Slight problems  Moderate problems  Severe problems  Extreme problems | 11 (6.0%)  35 (19.2%)  78 (42.9%)  49 (26.9%)  9 (4.9%) | 103 (28.7%)  109 (30.4%)  111 (30.9%)  35 (9.7%)  1 (0.3%) |  | 8 (4.4%)  42 (23.1%)  72 (39.6%)  52 (28.6%)  8 (4.4%) | 94 (26.2%)  114 (31.8%)  101 (28.1%)  40 (11.1%)  10 (2.8%) |
|  | **Anxiety/depression,  n (%)** | No problems  Slight problems  Moderate problems  Severe problems  Extreme problems | 27 (14.8%)  42 (23.1%)  71 (39.0%)  36 (19.8%)  6 (3.3%) | 233 (64.9%)  90 (25.1%)  32 (8.9%)  3 (0.8%)  1 (0.3%) |  | 34 (18.7%)  45 (24.7%)  62 (34.1%)  34 (18.7%)  7 (3.8%) | 227 (63.2%)  92 (25.6%)  30 (8.4%)  8 (2.2%)  2 (0.6%) |

Health-related quality of life as assessed by the EQ-5D-5L™ at baseline and follow-up differentiated by low/high New York Heart Association functional class and presence of psychosocial distress. PSD(–) = Psychosocial distress absent; PSD(+) = Psychosocial distress present; PSD classification according to hierarchical algorithm (for details see Methods).

*New York Heart Association functional class.

**Missing values in NYHA I/II group and PSD(–)= 4.6%; PSD(+): 4.8%; Missing values in NYHA III/IV group and PSD(–)= 4.2%; PSD(+): 5.5%.
