# Supplementary material for: Prognostic factors associated with quality of life in heart failure patients considering the use of the generic EQ-5D-5L™ in primary care: new follow-up results of the observational RECODE-HF study
Source: BMC Fam Pract. 2021 Oct 13;22:202. doi: 10.1186/s12875-021-01554-1 (PMC8515733; doi:10.1186/s12875-021-01554-1)
Supplement: Supplementary file 2 — Additional file 2: Table 4. Twelve-month change in health-related QoL by NYHA functional class and presence of psychosocial distress. Parametrisation of the twelve-month change in EQ-5D-5L elements differentiated by low/ high New York Heart Association functional class and presence of psychosocial distress. [file 12875_2021_1554_MOESM2_ESM.docx]

**Table 4.** Twelve-month change in health-related QoL by NYHA functional class and presence of psychosocial distress

| **EQ-5D-5L™ element** | **Parametrisation** | **NYHA functional class I/II, N=1779** | |  | **NYHA functional class III/IV, N=541** | |
| --- | --- | --- | --- | --- | --- | --- |
|  |  | **PSD(+)**  **N=439** | **PSD(–)**  **N=1340** |  | **PSD(+)**  **N=182** | **PSD(–)**  **N=359** |
| **VAS*** | Mean difference** (95% CI)  P-value | -0.76 (-2.56; 1.04)  0.407 | 1.97 (1.11; 2.84)  <0.001 |  | -1.30 (-4.60; 2.00)  0.439 | 3.20 (1.34; 5.10)  <0.001 |
| **German index** | Mean difference** (95% CI)  P-value | 0.015 (-0.002; 0.034)  0.080 | 0.021 (0.015; 0.028)  <0.001 |  | 0.017 (-0.014; 0.047)  0.289 | 0.032 (0.016; 0.048)  <0.001 |
| **Mobility** | OR (95% CI)  P-value  Increase*** | 13.53 (8.11; 22.58)  0.500  33.0% | 13.39 (10.28; 17.44)  <0.001  27.0% |  | 20.25 (5.32; 77.01)  0.791  50.0% | 30.07 (16.06; 56.28)  0.119  29.0% |
| **Self-care** | OR (95% CI) P-value  Increase*** | 21.98 (13.27; 36.41)  0.068  17.4% | 26.12 (17.88; 38.17)  <0.001  8.9% |  | 14.09 (6.52; 30.44)  0.311  35.6% | 22.10 (11.83; 41.28)  0.002  15.3% |
| **Usual activities** | OR (95% CI) P-value  Increase*** | 15.22 (8.50; 27.25)  0.064  34.2% | 12.33 (9.52; 15.97)  0.118  24.1% |  | 344.00 (31.05; 3811.37)  0.625  33.3% | 19.52 (11.12; 34.24)  0.130  21.7% |
| **Pain/discomfort** | OR (95% CI) P-value  Increase*** | 13.18 (6.44; 26.98)  0.392  57.1% | 9.17 (7.06; 11.91)  0.017  37.3% |  | 12.45 (2.52; 61.51)  0.581  72.7% | 14.53(8.28; 25.49)  0.321  35.9% |
| **Anxiety/depression** | OR (95% CI)  P-value  Increase*** | 12.28 (6.63; 22.74)  0.025  39.7% | 9.47 (7.24; 12.39)  0.162  16.9% |  | 11.07 (4.45; 27.54)  0.265  40.7% | 10.63 (6.41; 17.64)  0.576  18.5% |

Parametrisation of the twelve-month change in EQ-5D-5L elements differentiated by low/ high New York Heart Association functional class and presence of psychosocial distress. NYHA, New York Heart Association; QoL, quality of life; PSD, psychosocial distress classified according to hierarchical algorithm (Eisele et al. 2017); PSD(–) = Psychosocial distress absent; PSD(+) = Psychosocial distress present; PSD classification according to hierarchical algorithm (for details see Methods); VAS, visual analog scale; OR, odds ratio; CI, confidence interval. For calculation of P-values, paired t-tests were used for EQ VAS and index, and the McNemar test for the EQ-5D-5L™ items, respectively.

*Missing values in NYHA I/II group and PSD(–)= 4.6%; PSD(+): 4.8%; Missing values in NYHA III/IV group and PSD(–)= 4.2%; PSD(+): 5.5%. **As indicated by the 12-month minus the baseline VAS value. Negative values report a worsened actual well-being at the 12-month follow-up. ***Percent of patients reporting health-related quality of life problems at 12-month visit, but without respective problem at baseline (based on number of patients without health-related quality of life problems at baseline).
