# Supplementary material for: Prognostic factors associated with quality of life in heart failure patients considering the use of the generic EQ-5D-5L™ in primary care: new follow-up results of the observational RECODE-HF study
Source: BMC Fam Pract. 2021 Oct 13;22:202. doi: 10.1186/s12875-021-01554-1 (PMC8515733; doi:10.1186/s12875-021-01554-1)
Supplement: Supplementary file 3 — Additional file 3: Table 5. EQ-5D-5L™ ratings obtained after 12-month in RECODE-HF patients compared to the German population norm. Comparison of the twelve-month EQ-5D-5L™ ratings to German population norm values as assessed by the EQ-5D-3L (Szende, Janssen and Cabases 2014), age group 65–74 (n = 213) and 75+ (n = 149) reported, respectively, related to our RECODE-HF patient sample. [file 12875_2021_1554_MOESM3_ESM.docx]

**Table 5.** EQ-5D-5L™ ratings obtained after 12-month in RECODE-HF patients compared to the German population norm

|  |  |  | **RECODE-HF**  **study** | **German population norm*** | **P**  **value** |
| --- | --- | --- | --- | --- | --- |
| **Age group**  **below 75 years** | **EQ-5D-5L™, mean (95% CI)**  **N=1080** | VAS | 62.5 (61.2–63.7) | 68.6 | <0.001 |
|  |  | Index | 0.810 (0.798–0.821) | 0.838 | <0.001 |
|  | **EQ-5D-5L™  Self-reported problems* (%)**  **N=1112** | Mobility | 55.3 | 33.5 | <0.001 |
|  |  | Self-care | 17.8 | 5.5 | <0.001 |
|  |  | Usual activities | 54.2 | 19.2 | <0.001 |
|  |  | Pain/discomfort | 71.0 | 43.9 | <0.001 |
|  |  | Anxiety/depression | 44.2 | 4.7 | <0.001 |
| **Age group**  **above 75 years**  **n=** | **EQ-5D-5L™, mean (95% CI)**  **N=1088** | VAS | 55.9 (54.7–57.2) | 60.5 | <0.001 |
|  |  | Index | 0.729 (0.715–0.743) | 0.771 | <0.001 |
|  | **EQ-5D-5L™  Self-reported problems* (%)**  **N=1125** | Mobility | 73.6 | 54.2 | <0.001 |
|  |  | Self-care | 39.0 | 16.0 | <0.001 |
|  |  | Usual activities | 70.1 | 33.8 | <0.001 |
|  |  | Pain/discomfort | 79.9 | 52.3 | <0.001 |
|  |  | Anxiety/depression | 46.2 | 6.6 | <0.001 |

Comparison of the twelve-month EQ-5D-5L™ ratings to German population norm values as assessed by the EQ-5D-3L (Szende, Janssen and Cabases 2014), age group 65-74 (n = 213) and 75+ (n = 149) reported, respectively, related to our RECODE-HF patient sample.*Includes slight, moderate, severe, or extreme problems. P-values derived using one-sample t-tests for EQ VAS and German EQ index, and chi-square test for individual EQ-5D-5L™ items, respectively.
